# Prediction Model of Acute Respiratory Failure in Patients with Acute Pesticide Poisoning by Intentional Ingestion: Prediction of Respiratory Failure in Pesticide Intoxication (PREP) Scores in Cohort Study

**DOI:** 10.3390/jcm11041048

**Published:** 2022-02-17

**Authors:** Nam-Jun Cho, Samel Park, Jiwon Lyu, HwaMin Lee, Min Hong, Eun-Young Lee, Hyo-Wook Gil

**Affiliations:** 1 Department of Internal Medicine, Soonchunhyang University Cheonan Hospital, Cheonan 31151, Korea; chonj@schmc.ac.kr (N.-J.C.); samelpark17@schmc.ac.kr (S.P.); 78214@schmc.ac.kr (J.L.); eylee@schmc.ac.kr (E.-Y.L.); 2 Department of Computer Software Engineering, Soonchunhyang University, Asan 31538, Korea; leehm@sch.ac.kr (H.L.); mhong@sch.ac.kr (M.H.)

**Keywords:** poisoning, pesticides, artificial respiration, triage, respiratory insufficiency

## Abstract

Acute respiratory failure is the primary cause of mortality in patients with acute pesticide poisoning. The aim of the present study was to develop a new and efficient score system for predicting acute respiratory failure in patients with acute pesticide poisoning. This study was a retrospective observational cohort study comprised of 679 patients with acute pesticide poisoning by intentional poisoning. We divided this population into a ratio of 3:1; training set (*n* = 509) and test set (*n* = 170) for model development and validation. Multivariable logistic regression models were used in developing a score-based prediction model. The Prediction of Respiratory failure in Pesticide intoxication (PREP) scoring system included a summation of the integer scores of the following five variables; age, pesticide category, amount of ingestion, Glasgow Coma Scale, and arterial pH. The PREP scoring system developed accurately predicted respiratory failure (AUC 0.911 [0.849−0.974], positive predictive value 0.773, accuracy 0.873 in test set). We came up with four risk categories (A, B, C and D) using PREP scores 20, 40 and 60 as the cut-off for mechanical ventilation requirement risk. The PREP scoring system developed in the present study could predict respiratory failure in patients with pesticide poisoning, which can be easily implemented in clinical situations. Further prospective studies are needed to validate the PREP scoring system.

## 1. Introduction

Pesticides are a large and heterogeneous group of chemicals which include insecticides, herbicides, and fungicides meant to control pests. Unfortunately, along with their advantages, pesticides poisoning is a major public health concern worldwide. While pesticides are characterized by various degrees of toxicity, high mortality is a common feature with all types of pesticide poisoning [1,2]. A majority of the deaths result from different complications including arrhythmia, hypotension, acid-base disturbance, and respiratory failure [3,4,5]. Among them, respiratory failure is the primary cause of mortality, although the clinical features of each pesticide poisoning case might be different depending on the chief ingredient of the pesticide ingested. Among the types of pesticide poisoning, the relationships of organophosphate and carbamate poisoning with respiratory failure have been widely studied [6,7,8] even though respiratory failure is also observed in patients with other pesticide poisoning.

Acute respiratory failure is one of the most common acute organ failures in hospitals [9] and is associated with a six-month mortality in 30% of the patients, increased hospital readmission, and functional impairment among survivors [10]. Clinical deterioration in acute respiratory failure can be seen 8–48 h prior to critical care intervention [11]. Respiratory failure is an important predictor of mortality in pesticide poisoning. Failure to identify developing respiratory failure is the most common reason for delayed resuscitation, which has been associated with increased hospital mortality. Clinically, it is very important to thoroughly evaluate the patient’s condition for possible exacerbation, because even with mild poisoning symptoms at admission, severe symptoms including respiratory failure may develop later [2]. Therefore, a model that can predict respiratory failure is essential, as has been for pneumonia [12]. It is hard to develop an efficient model to predict respiratory failure in patients with acute pesticide poisoning because the mechanisms of respiratory failure induced by pesticide poisoning vary depending on the specific pesticide class. There is a need for a model that can predict respiratory failure in patients with acute pesticide poisoning, although this model could not reveal the mechanism of respiratory failure according to pesticide class.

The objective of our study was to investigate a prediction model for respiratory failure in patients with acute pesticide poisoning.

## 2. Materials and Methods

### 2.1. Study Population and Study Design

**Figure 1:**
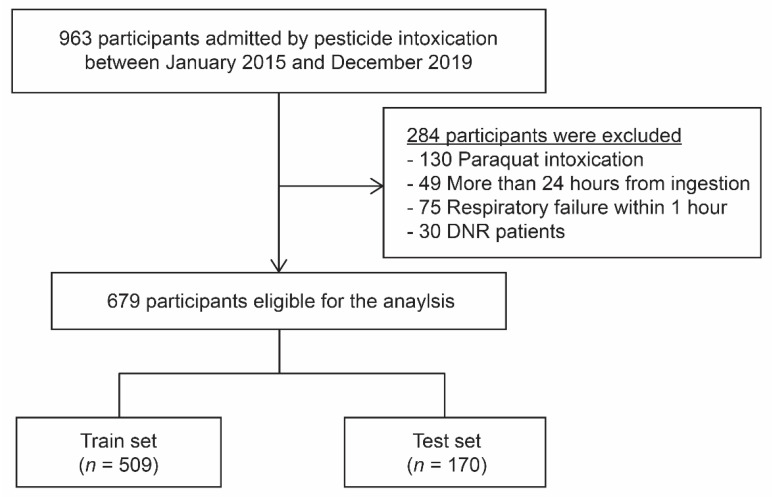
Flow diagram of the study inclusion and exclusion criteria.

We conducted a retrospective observational cohort study at Soonchunhyang University Cheonan Hospital between January 2015 and December 2019. A total of 963 pesticide intoxication patients aged 19 years or older were admitted to the Institute of Pesticide Poisoning at Soonchunhyang University Cheonan Hospital. We excluded the patients with paraquat poisoning (because of the high fatality rate), those who visited the hospital more than 24 h after pesticide ingestion, and those diagnosed with respiratory failure within one hour from hospital arrival. Do-Not-Resuscitate (DNR) patients denying mechanical ventilators were also excluded (Figure 1). A total of 679 patients with acute pesticide poisoning by intentional poisoning were enrolled. The entire cohort was randomly divided into two groups; a training and test dataset at an approximate 3:1 ratio. The present study was reviewed and approved by the Soonchunhyang University Cheonan Hospital’s Investigational Review Board (IRB number: 2020-02-016). The requirement for informed consent was waived because of the retrospective design of the study. This study was conducted in accordance with the Declaration of Helsinki.

### 2.2. Data Collection and Processing

Patients’ demographic features were acquired from the electronic medical records system and were recorded by the physicians on standardized data collection forms. The exact time of the patient’s pesticide exposure and arrival at the hospital was investigated by reviewing the emergency room chart. The amount of pesticide ingested was estimated from the number of swallowing, where one mouthful was considered to be 20 mL. We collected laboratory data for test whose final results were reported within the first one hour of admission, such as arterial blood gas analysis results and blood lactate levels. The continuous data, including age, body mass index, Glasgow Coma Scale, the amount of ingestion, vital signs, and laboratory data, were categorized for integration into a scoring system. The categorical data were cleaned and transformed into a set of binary variables (dummy variable encoding).

### 2.3. Study Outcome

The study outcome was a need for mechanical ventilation (MV) within three days after ingestion of pesticide [13,14]. We set three days starting from the ingestion time as the monitoring period of the outcomes because most of the events (96.0%) have been reported to occur within three days. Furthermore, if the window period was extended, non-pesticide-related factors could influence the respiratory problems. Cases of non-invasive ventilation and endotracheal intubation without a ventilator were not included in this outcome. For the survival analyses we included time-event data of whether and when the outcome occurred.

### 2.4. Variable Selection

Candidate variables associated with ventilator requirement in the univariable logistic regression analysis (*p* < 0.1) were chosen, and additional variable selection processes were conducted using three methods: stepwise method, best subset method, and LASSO regression method [15,16,17]. We used stepwise approach starting with the global model, cycling between backward elimination and forward selection steps until convergence using Akaike information criterion. The best subset method selected variables from all possible subset models according to Bayesian information criterion, using “leaps” package in R software. Regularized regression with LASSO penalties was also conducted for variable selection using “glmnet” package in R software.

### 2.5. Prediction Model Construction and Scoring System

Multivariable logistic regression models were used to develop a score-based prediction model. We compared three logistic regression models made by the three different variable selection methods described above. The Receiving Operating Characteristic (ROC) curve was used as a metric to measure the prediction model performance. The area under ROC curve (AUC) of each of the prediction models was pairwise-compared using the DeLong test [18]. After choosing the final model, we multiplied the coefficients to set the highest sum of the model coefficients in the training dataset as 100 and rounded each coefficient to an integer to produce the scoring system. We evaluated the model calibration by visual inspection with calibration plots and by Hosmer-Lemeshow test [19].

### 2.6. Statistical Analysis

The statistical analyses were performed using R version 4.0.0 (The R Foundation for Statistical Computing, Vienna, Austria). The categorical variables are expressed as counts (percentage). The normally distributed continuous variables are expressed as means ± SD, and the non-normally distributed continuous variables are presented as medians (interquartile ranges). Two groups were compared with Student’s two-tailed unpaired t-test or Mann-Whitney U test, as appropriate. Pearson’s Chi-squared tests were used when comparing the categorical variables. Kaplan-Meier curve analysis was used to assess the association between the risk factors and the requirement of mechanical ventilation. A *p*-value less than 0.05 was considered statistically significant.

## 3. Results

### 3.1. Characteristics of Study Subjects

A total of 963 pesticide intoxication patients were admitted between January 2015 and December 2019. After the exclusion of 284 patients, 679 patients were selected for model construction (Figure 1). We divided this population into two groups at a 3:1 ratio, the training set (*n* = 509) and test set (*n* = 170), for model development and validation. Baseline characteristics of the study population are presented in Table 1. There was no statistical difference in clinical variables between the subject in the training set and those in the test set. The missing value counts of each variable in training and test set are presented in Table S1.

**Figure 2:**
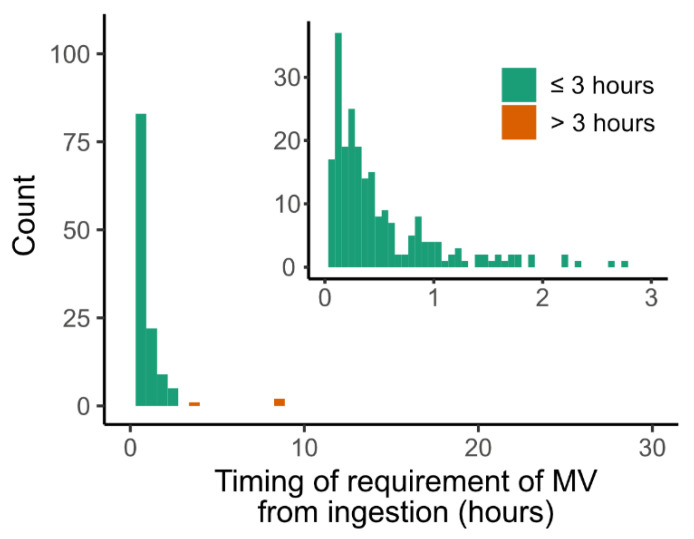
The timing of requirement of mechanical ventilation after pesticide ingestion. The observation period was 30 days in the larger plot and three days in the smaller plot (top-right position). MV, mechanical ventilation.

Most of the MV events were within the first three days after the pesticide ingestion (96% of total MV events) (Figure 2). The number of patients who required MV within three days was 95 (18.7%) in the training set and 33 (19.4%) in the test set. The percentage and timing of MV requirement according to the pesticide category are presented in Table S2. The percentages of organophosphate/carbamate (42.4%) and glufosinate (40.4%) intoxication were significantly higher than those of the other pesticides (7.0%). The median timing of MV requirement from ingestion in glufosinate intoxication was 16.0 (10.3–24.7) hours, which was late compared with those in other pesticides (7.8 (5.2–18.8) hours, *p* < 0.001), including organophosphate and carbamate (9.8 (5.8–20.3), *p* < 0.001).

### 3.2. Variable Selection

We conducted univariable logistic regression analysis in the training set to choose candidate variables that were used for developing a prediction model for respiratory failure in pesticide intoxication (PREP). Candidate variables included systolic BP, respiratory rate, body temperature, age, alcohol history, vomiting, amount of ingestion, pesticide category, Glasgow Coma Scale, arterial pH, and arterial pO_2_ (Table S3). The number of variables reduced after additional variable selection processes. We made three multivariable logistic regression models, Model 1, 2, and 3, using stepwise methods, best subset method, and LASSO regression method, respectively. The variables included in all of these models were age, amount of ingestion, pesticide category, Glasgow Coma Scale, and arterial pH. In addition to these variables, Model 1 used systolic BP and alcohol history, and Model 3 used respiratory rate. The three models’ variables and their estimates are presented in Table S4.

### 3.3. Selection of the Final Prediction Model and Building a Scoring System

**Figure 3:**
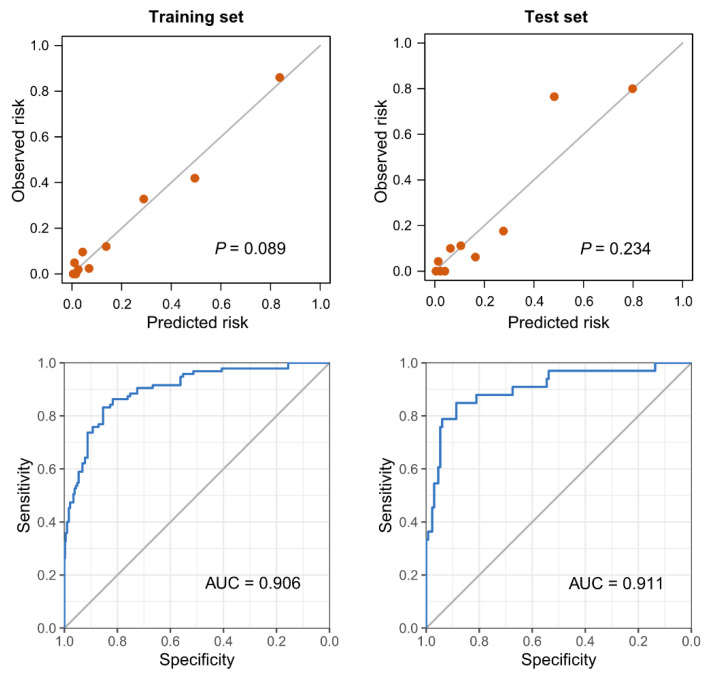
Calibration plots and receiver operating characteristics curves for the scoring model, predicting the requirement of mechanical ventilation. The *p*-values in the calibration plots were calculated by the Hosmer-Lemeshow test to assess the goodness of fit.

We compared the three logistic regression models using AUC values, and sensitivity and positive predictive value (PPV) in both the training set and the test set (Table 2). There was no statistical difference of AUC values between the three models (*p*-value for Model 1 versus Model 2 = 0.598, Model 2 versus Model 3 = 0.245, Model 1 versus Model 3 = 0.364). We selected Model 2 as the final prediction model because Model 2 had excellent predicting ability despite having the least number of variables. Then, we made a scoring system by multiplying the coefficients of Model 2 by 9.1827 to make the highest score a hundred (Table 3). The discrimination and calibration results after converting the model coefficients to integer scores are presented in Figure 3.

### 3.4. Selection of the Score Threshold

**Figure 4:**
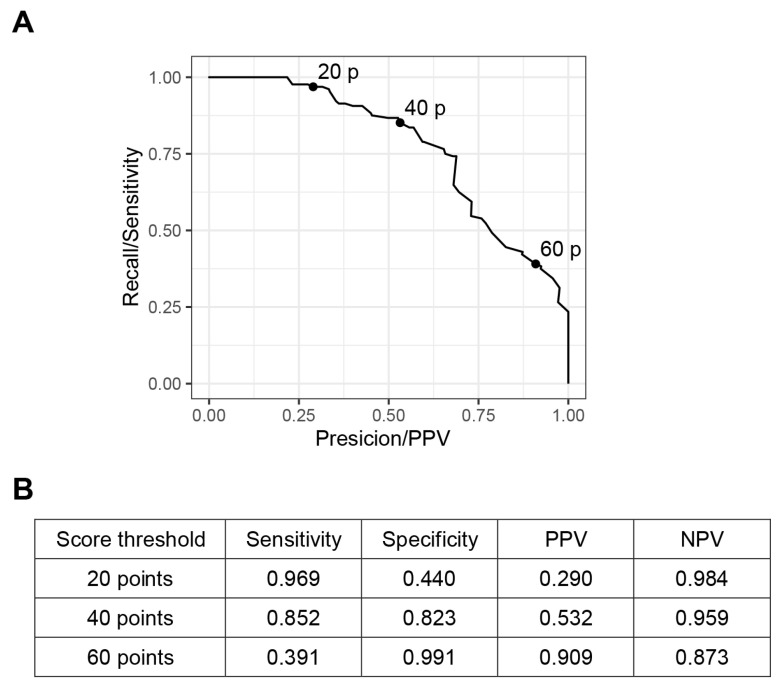
Prediction performance illustrated by a precision-recall curve with the score threshold. (**A**) The graph shows the precision-recall curve for the risk of the mechanical ventilation requirement. The precision (positive predictive value) and recall (sensitivity) values of the score threshold 20, 40, and 60 are presented as dots (20 p, 40 p, and 60 p). (**B**) The exact values of sensitivity and specificity, positive predictive value, and negative predictive value according to the score thresholds are presented. PPV, positive predictive value; NPV negative predictive value.

After building the prediction model, we selected the score threshold that was more convenient to use in the scoring system (Figure 4). The best cut-off score which maximized the sum of sensitivity and specificity was 42 (sensitivity 0.836, specificity 0.851), so we used score 40 as one of score thresholds. The sensitivity at the score threshold 20 was very high (sensitivity 0.969, PPV 0.290), so if the prediction score is under 20, the probability of progressing to mechanical ventilation requirement status is very low. On the other hand, a score threshold of 60 has very high PPV (sensitivity 0.391, PPV 0.909), so if the prediction score is higher than 60, it is very likely to require mechanical ventilation. Using scores 20, 40, and 60 as the cut-off, we made risk categories A, B, C, and D. The incidences of MV requirement according to the four risk categories in the training and the test set are presented in Table 4.

### 3.5. Survival Analyses to Estimate the Risk of MV Requirement

We also conducted survival analyses which estimated the probability of MV requirement. Kaplan-Meier curves that present the probability of the event at a certain time interval according to each predictor variable were made (Figure S1). In the pesticides categories analysis, the organophosphate and carbamate group, as well as the glufosinate group, had a similar probability of the event. For that reason, organophosphate, carbamate, and glufosinate groups were combined as one category in the predictive models. However, respiratory failure after poisoning in the glufosinate group occurred later than in the organophosphate and carbamate group (median time 16.0 (10.3–24.7) hours in the glufosinate group versus 9.8 (5.8–20.3) hours in the organophosphate and carbamate group). Analyses of the other predictors, including age, amount of ingestion, GCS score, and arterial pH, showed significant differences in the probability of the event development between each category. Kaplan-Meier curve for the risk category from the prediction model is presented in Figure S2. The curve shows good discrimination between the categories.

## 4. Discussion

Our study results showed that our PREP scoring system could accurately predict respiratory failure requiring mechanical ventilation in patients with acute pesticide poisoning. We developed a simple individual risk score to identify progression of acute respiratory failure in acute pesticide poisoning at the time of hospitalization individuals. Based on our findings, five variables are enough for a PREP scoring system. Scores higher than 40 suggest a high possibility of respiratory failure. This could help clinicians to predict deterioration of pesticide poisoned patients and identify patients who need to be admitted to the intensive care unit.

Examination at the initial stages of acute pesticide poisoning may not show severe symptoms, however serious complications could develop later [20,21]. Since respiratory failure is the primary cause of mortality in pesticide poisoning, the PREP scoring system is important to predict acute respiratory failure in patients who have no symptoms at the initial stages. Our study showed that most cases of acute respiratory failure developed within the first day of poisoning (Figure 2). We recommend that patients who have high scores (more than 40) should be carefully observed during the first 24 h.

Alapat et al. reported that the management of toxicity in critical care requires thorough evaluation to enable clinicians to come up with focused therapies [22]. In our study, 19% of the patients had respiratory failure requiring MV within three days. These findings are very important in the management of pesticide poisoning patients. Clinicians could underestimate the risk of pesticide poisoning patients due to lack of experience, proper guidelines, or a scoring system. The PREP scoring system is expected to be helpful in identifying patients who need more advanced care.

Pesticide poisoning is characterized by high mortality compared with other poisonings [23], but fatality of agricultural pesticides after self-poisoning pesticide poisoning depends on the class of pesticides [24]. There are some studies on mortality prediction in acute pesticide poisoning [3,4,25]. In common clinical situations, scoring systems for patients in intensive care unit have been developed and introduced during the last over 30 years [26,27]. The Acute Physiology and Chronic Health Evaluation score and the Simplified Acute Physiology Score are probably the best-known and most widely used score systems in ICU patients. Although these scoring systems has been tested in acute pesticide poisoning [2,5], they do not reflect the characteristics of patients with acute pesticide poisoning. Commendably, the PREP scoring system developed in the present study included the clinical factors of acute pesticide poisoning.

Clinical features in pesticide poisoning vary across different types of pesticides. Respiratory failure has been reported as a common complication in organophosphate and carbamate poisoning [7,8]. The pathophysiology of organophosphate and carbamate-induced respiratory failure can be explained by three mechanisms: depression of central respiratory drive from the respiratory center in the ventrolateral medulla, weakness of the muscles of respiration, and organophosphate-induced bronchospasm induced via local and vagal mechanisms [7]. In addition, in the present study, glufosinate ingestion was identified as an important risk factor for acute respiratory failure; the incidence rate of respiratory failure in glufosinate poisoning is the same as that of organophosphate and carbamate. Intriguingly, given the comparable incidence of respiratory failure, patients who were intoxicated with organophosphate and carbamate showed respiratory acidosis at emergency room, but this was not observed in glufosinate poisoning [4]. In this study, it was revealed that the development of respiratory failure in glufosinate poisoning was slightly delayed (6 h later) as compared to organophosphate and carbamate, therefore, respiratory failure was not captured at the emergency department in cases of glufosinate intoxication. The exact mechanism of respiratory failure in glufosinate poisoning has not been fully revealed. We previously reported that brain glucose metabolism decreased in patients with glufosinate intoxication [28]. Also, Park et al. reported that the inhibition of both glycolysis and mitochondrial respiration pathway might be associated with oxidative stress and ferroptosis in human astrocytes [29]. Given the role of astrocytes in glucose metabolism and putative mechanisms associated with glufosinate [28], respiratory failure in glufosinate intoxication might be attributed to impairment of glucose metabolism. We hope further study could reveal the mechanism of respiratory failure in glufosinate poisoning.

Our study has some limitations. First, our data was from a single center, and our scoring system was not externally validated. The differences in the types of pesticide poisoning and patient characteristics in other centers can affect the scoring system accuracy. Second, since the study had a retrospective design, most of the information relied on reviews of health records. The amount and ingestion time of pesticide was mainly based on the description of patients or their relatives. Prospective validation studies may confirm the accuracy and utility of our model.

## 5. Conclusions

We developed and suggested a PREP scoring system for predicting acute respiratory failure in patients with acute pesticide poisoning, which is useful in the management pesticide poisoning. We also demonstrated that glufosinate poisoning is important risk factor for acute respiratory failure.

## Figures and Tables

**Table 1:** Baseline characteristics of study participants.

	All Patients (*n* = 679)	Training Set (*n* = 509)	Test Set (*n* = 170)	*p*-Value
Age, years	61.2 ± 16.0	61.1 ± 16.1	61.6 ± 15.5	0.711
Sex, male (%)	429 (63.2)	322 (63.3)	107 (62.9)	1.000
BMI, kg/m^2^	22.9 ± 3.3	22.9 ± 3.3	22.8 ± 3.6	0.715
Alcohol history, yes (%)	327 (48.2)	248 (48.7)	79 (46.5)	0.736
Diabetes, present (%)	123 (18.1)	91 (17.9)	32 (18.8)	0.888
Hypertension, present (%)	241 (35.5)	185 (36.3)	56 (32.9)	0.457
Lung disease, present (%)	59 (8.7)	43 (8.4)	16 (9.4)	0.830
Cardiac disease, present	42 (6.2)	31 (6.1)	11 (6.5)	1.000
Time to hospital presentation after ingestion, hours	2.97 (1.77, 5.00)	2.97 (1.82, 5.00)	2.98 (1.76, 5.01)	0.537
Pesticide category				0.348
Glufosinate	151 (22.2)	110 (21.6)	41 (24.1)	
Glyphosate	186 (27.4)	146 (28.7)	40 (23.5)	
OP or CM	85 (12.5)	66 (13.0)	19 (11.2)	
Pyrethroid	69 (10.2)	46 (9.0)	23 (13.5)	
Other pesticides	188 (27.7)	141 (27.7)	47 (27.6)	
Amount of ingestion				0.837
Under 100 cc	285 (42.0)	209 (41.1)	76 (44.7)	
100–300 cc	246 (36.2)	186 (36.5)	60 (35.3)	
Over 300 cc	80 (11.8)	61 (12.0)	19 (11.2)	
Unknown	68 (10.0)	53 (10.4)	15 (8.8)	
Systolic BP, mmHg	133.9 ± 25.9	134.2 ± 25.8	133.0 ± 26.5	0.606
Diastolic BP, mmHg	78.5 ± 14.4	78.7 ± 13.8	77.9 ± 16.1	0.561
Pulse rate, beats/min	88.0 ± 16.4	87.9 ± 15.7	88.2 ± 18.5	0.870
RR, breaths/min	19.4 ± 2.9	19.4 ± 3.0	19.4 ± 2.3	0.996
Body temperature, °C	36.4 ± 0.6	36.4 ± 0.6	36.4 ± 0.7	0.495
Glasgow Coma Scale	15.0 (14.0, 15.0)	15.0 (14.0, 15.0)	15.0 (13.8, 15.0)	0.671
Gastric lavage, yes (%)	419 (61.7)	318 (62.5)	101 (59.4)	0.495
Vomiting, yes (%)	259 (38.1)	196 (38.5)	63 (37.1)	0.806
Arterial pH	7.37 ± 0.09	7.38 ± 0.09	7.37 ± 0.09	0.560
pCO_2_, mmHg	37.1 ± 7.8	37.2 ± 7.7	36.6 ± 7.9	0.383
pO_2_, mmHg	87.1 ± 28.4	86.9 ± 27.4	87.8 ± 31.3	0.743
HCO_3_^−^, mmol/L	22.5 ± 3.9	22.6 ± 3.9	22.3 ± 4.0	0.482

Data are presented as mean ± SD, median (interquartile range), or count (%) as appropriate. Other pesticides include acetanilide, acetylaniline, alryoxylcarboxide, amide, anilin, arsenic, (aryloxy)phenopropionate, benzohydrazide, benzoate, chlorfenapyr, chloroacetamide, chloronicotinyl, diamide, diazine, dinitroaniline, endosulfan, fungicide, insect growth regulator, lambda cyhalothrin, neonicotinoid, niacin, oxadiazole, phenoxy, pyrol, sulfonylurea, sulfoximine, sulfuryl fluoride, tetramic acid, tetrazolium oxide, urea, and unknown pesticides. Gastric lavage refers to cases where gastric lavage was performed at another hospital or after visiting this hospital. Vomiting refers to cases of vomiting before visiting this hospital. BMI, body mass index; OP, organophosphate; CM, carbamate; BP, blood pressure; RR, respiratory rate.

**Table 2:** Comparison of the prediction models for the requirement of mechanical ventilation.

	Training Set				Test Set			
	Sensitivity	PPV	Accuracy	AUC (95% CI)	Sensitivity	PPV	Accuracy	AUC (95% CI)
Model 1	0.600	0.750	0.886	0.914 (0.882–0.946)	0.606	0.833	0.895	0.912 (0.847–0.976)
Model 2	0.537	0.739	0.878	0.905 (0.871–0.940)	0.515	0.773	0.873	0.911 (0.849–0.974)
Model 3	0.537	0.761	0.881	0.907 (0.873–0.942)	0.515	0.800	0.879	0.915 (0.853–0.977)

PPV, positive predictive value; AUC, area under the Receiving Operating Characteristic curve.

**Table 3:** Prediction of Respiratory failure in Pesticide intoxication (PREP) in patients with acute pesticide intoxication using scoring system.

Variables	Scores
Age ≤50	0
Age 50–70	11
Age >70	15
Pesticide category, OP, CM, or GF, versus other pesticides	25
Amount of ingestion, ≤100 cc	0
Amount of ingestion, 100–300 cc	8
Amount of ingestion, >300 cc	9
Amount of ingestion, unknown	16
Glasgow Coma Scale, >12	0
Glasgow Coma Scale, 8–12	11
Glasgow Coma Scale, ≤8	26
Arterial pH, >7.35	0
Arterial pH, 7.25–7.35	6
Arterial pH, ≤7.25	18

OP, organophosphate; CM, carbamate; GF, glufosinate.

**Table 4:** Risk categories according to the risk scores.

PREP Category	Total Score	Description	Event Occurrence (%)	
			Training Set	Test Set
A	0–19	Less likely	1.58%	1.89%
B	20–39	Possible	7.27%	6.73%
C	40–59	Likely	38.5%	41.5%
D	60–100	Very likely	90.5%	92.3%

## Data Availability

The datasets used and/or analyzed during the current study are available from the corresponding author upon reasonable request.

## Supplementary Materials

The following supporting information can be downloaded at: https://www.mdpi.com/article/10.3390/jcm11041048/s1. Table S1: The missing value counts of each variable in the training and test set; Table S2: Percentage and timing of mechanical ventilation requirements according to the pesticide category; Table S3: Univariable logistic regression for predicting the requirements of MV; Table S4: Multivariable logistic regression models for predicting the requirements of MV; Figure S1: Kaplan-Meier curves for the mechanical ventilation requirements according to the categories of each predictor; Figure S2: Kaplan-Meier curve for the mechanical ventilation requirement according to the risk categories of the prediction model.
