# Supplementary material for: Prediction Model of Acute Respiratory Failure in Patients with Acute Pesticide Poisoning by Intentional Ingestion: Prediction of Respiratory Failure in Pesticide Intoxication (PREP) Scores in Cohort Study
Source: J Clin Med. 2022 Feb 17;11(4):1048. doi: 10.3390/jcm11041048 (PMC8875988; doi:10.3390/jcm11041048)
Supplement: Supplementary file 1 [file jcm-11-01048-s001.zip › jcm-1576489-supplementary.pdf]

Table S1. The missing value counts of each variable in the training and test set.

	**All patients (*n* = 679)**	**Training set (*n* = 509)**	**Test set (*n* = 170)**
Age	0	0	0
Sex	0	0	0
Body mass index	48 (7.1%)	31 (6.1%)	17 (10.0%)
Alcohol history	12 (1.8%)	8 (1.6%)	4 (2.4%)
Diabetes	2 (0.3%)	2 (0.4%)	0
Hypertension	2 (0.3%)	2 (0.4%)	0
Lung disease	2 (0.3%)	2 (0.4%)	0
Cardiac disease	2 (0.3%)	2 (0.4%)	0
Time to hospital presentation after ingestion, hours	0	0	0
Pesticide category	0	0	0
Amount of ingestion	0	0	0
Systolic blood pressure	0	0	0
Diastolic blood pressure	0	0	0
Pulse rate	0	0	0
Respiratory rate	0	0	0
Body temperature	0	0	0
Glasgow Coma Scale	4 (0.6%)	2 (0.4%)	2 (1.2%)
Gastric lavage	6 (0.9%)	5 (1.0%)	1 (0.6%)
Vomiting	0	0	0
Arterial pH	4 (0.6%)	1 (0.2%)	3 (1.8%)
pCO_2	4 (0.6%)	1 (0.2%)	3 (1.8%)
pO_2	4 (0.6%)	1 (0.2%)	3 (1.8%)
HCO_3^-	5 (0.7%)	1 (0.2%)	4 (2.4%)

Table S2. Percentage and timing of mechanical ventilation requirements according to the pesticide category

**Pesticide category**	**Total number**	**Requirement of MV (%)**	**Timing of MV requirement (hour)**
OP and CM	85	36 (42.4%)	9.8 (5.8–20.3)
Glufosinate	151	61 (40.4%)	16.0 (10.3–24.7)
Glyphosate	186	14 (7.5%)	9.3 (5.7–20.7)
Pyrethroid	69	1 (1.4%)	7.8 (7.8–7.8)
Other pesticides	188	16 (8.5%)	5.4 (3.7–7.8)

The timings of the mechanical ventilation requirements are presented as median (interquartile range). MV, mechanical ventilation; OP, organophosphate; CM, carbamate.

Table S3. Univariable logistic regression for predicting the requirements of MV

	**Odd ratio**	**95% CI**	***P*-value**
Age ≤50	Reference		
Age 50–70	2.60	1.28–5.73	0.012
Age >70	4.83	2.44–10.47	<0.001
Sex, male versus female	0.72	0.46–1.13	0.151
BMI ≤18.5 kg/m^2	Reference		
BMI 18.5–25 kg/m^2	0.74	0.36–1.67	0.445
BMI 25–30 kg/m^2	0.62	0.26–1.53	0.284
BMI >30 kg/m^2	0.44	0.02–2.95	0.471
Alcohol history, yes versus no	0.83	0.33–0.83	0.007
Diabetes, present versus absent	0.91	0.49–1.61	0.755
Hypertension, present versus absent	1.41	0.89–2.22	0.135
Lung disease, present versus absent	1.78	0.85–3.52	0.111
Cardiac disease, present versus absent	1.56	0.63–3.46	0.301
Pesticide category, OP, CM or GF versus other pesticides	10.02	6.01–17.269	<0.001
Amount of ingestion, ≤100 cc	Reference		
Amount of ingestion, 100–300 cc	3.41	1.88–6.48	<0.001
Amount of ingestion, >300 cc	5.05	2.39–10.81	<0.001
Amount of ingestion, unknown	7.31	3.46–15.76	<0.001
Systolic BP, 100–140 mmHg	Reference		
Systolic BP, ≤100 mmHg	1.09	0.47–2.29	0.825
Systolic BP, >140 mmHg	1.94	1.19–3.13	0.007
Diastolic BP, 40–90 mmHg	Reference		
Diastolic BP, ≤40 mmHg	1.51	0.07–11.93	0.724
Diastolic BP, >90 mmHg	1.31	0.65–2.47	0.430
Pulse rate, 60–100 beats/min	Reference		
Pulse rate, ≤60 beats/min	1.74	0.47–5.24	0.357
Pulse rate, >100 beats/min	1.45	0.82–2.49	0.185
Respiratory rate, 12–20 breaths/min	Reference		
Respiratory rate, ≤12breaths/min	22.26	5.43–149.82	>0.001
Respiratory rate, >20 breaths/min	2.08	1.20–3.51	0.007
Body temperature, 35–37.5 °C	Reference		
Body temperature, ≤35 °C	4.34	1.89–11.90	0.001
Body temperature, >37.5 °C	0.86	0.13–3.28	0.847
Glasgow Coma Scale, >12	Reference		
Glasgow Coma Scale, 8–12	6.49	3.48–12.05	<0.001
Glasgow Coma Scale, ≤8	22.52	10.24–53.83	<0.001
Gastric lavage, yes versus no	0.84	0.53–1.35	0.467

Vomiting, yes versus no	1.65	1.05–2.59	0.029
Arterial pH, >7.35	Reference		
Arterial pH, 7.25–7.35	2.16	1.28–3.62	0.004
Arterial pH, ≤7.25	10.31	5.08–21.46	<0.001
pCO_2, ≤60 versus >60 mmHg	1.08	0.67–1.71	0.760
pO_2, >40 versus ≤40 mmHg	2.44	1.56–5.33	0.029

BMI, body mass index; OP, organophosphate; CM, carbamate; GF, glufosinate; BP, blood pressure.

Table S4. Multivariable logistic regression models for predicting the requirements of MV

	Model 1 (Stepwise)		Model 2 (Best subset)		Model 3 (LASSO)	
	**Estimate (95% CI)**	***P*-value**	**Estimate (95% CI)**	***P*-value**	**Estimate (95% CI)**	***P*-value**
Age ≤50	Reference		Reference		Reference	
Age 50–70	1.10 (0.13–2.08)	0.027	1.18 (0.24–2.13)	0.014	1.14 (0.19–2.09)	0.018
Age >70	1.29 (0.32–2.26)	0.009	1.66 (0.74–2.58)	<0.001	1.59 (0.67–2.51)	0.001
Alcohol history, yes versus no	-0.61 (-1.30–0.08)	0.081	–	–	–	–
Pesticide category, OP, CM or GF versus other pesticides	2.72 (2.01–3.44)	<0.001	2.74 (2.05–3.44)	<0.001	2.73 (2.02–3.43)	<0.001
Amount of ingestion, ≤100 cc	Reference		Reference		Reference	
Amount of ingestion, 100–300 cc	2.82 (1.29–6.14)	0.009	0.93 (0.17–1.69)	0.017	0.92 (0.15–1.68)	0.019
Amount of ingestion, >300 cc	3.03 (1.13–8.13)	0.027	0.97 (0.00–1.94)	0.049	0.86 (-0.14–1.85)	0.091
Amount of ingestion, unknown	6.04 (2.16–16.9)	0.001	1.71 (0.71–2.71)	0.001	1.70 (0.70–2.70)	0.001
Systolic BP, 100–140 mmHg	Reference		–	–	–	–
Systolic BP, ≤100 mmHg	0.92 (0.29–2.93)	0.886	–	–	–	–
Systolic BP, >140 mmHg	2.04 (1.02–4.06)	0.043	–	–	–	–
Respiratory rate, 12–20 breaths/min	–	–	–	–	Reference	
Respiratory rate, >20 breaths/min	–	–	–	–	0.35 (-0.37–1.07)	0.342
Respiratory rate, ≤12breaths/min	–	–	–	–	1.47 (-0.85–3.80)	0.215
Glasgow Coma Scale, >12	Reference		Reference		Reference	
Glasgow Coma Scale, 8–12	1.20 (0.38–2.02)	0.004	1.24 (0.43–2.05)	0.003	1.33 (0.51–2.15)	0.003
Glasgow Coma Scale, ≤8	2.78 (1.69–3.87)	<0.001	2.83 (1.78–3.88)	<0.001	2.76 (1.68–3.83)	<0.001
Arterial pH, >7.35	Reference		Reference		Reference	
Arterial pH, 7.25–7.35	0.73 (0.04–1.42)	0.039	0.64 (-0.03–1.31)	0.063	0.62 (-0.06–1.30)	0.063
Arterial pH, ≤7.25	2.13 (1.09–3.18)	<0.001	1.95 (0.95–2.95)	<0.001	1.78 (0.74–2.82)	<0.001

The variables in Model 1, 2, and 3 were selected by stepwise methods, best subset method, and LASSO regression method, respectively. OP, organophosphate; CM, carbamate; GF, glufosinate; BP, blood pressure.

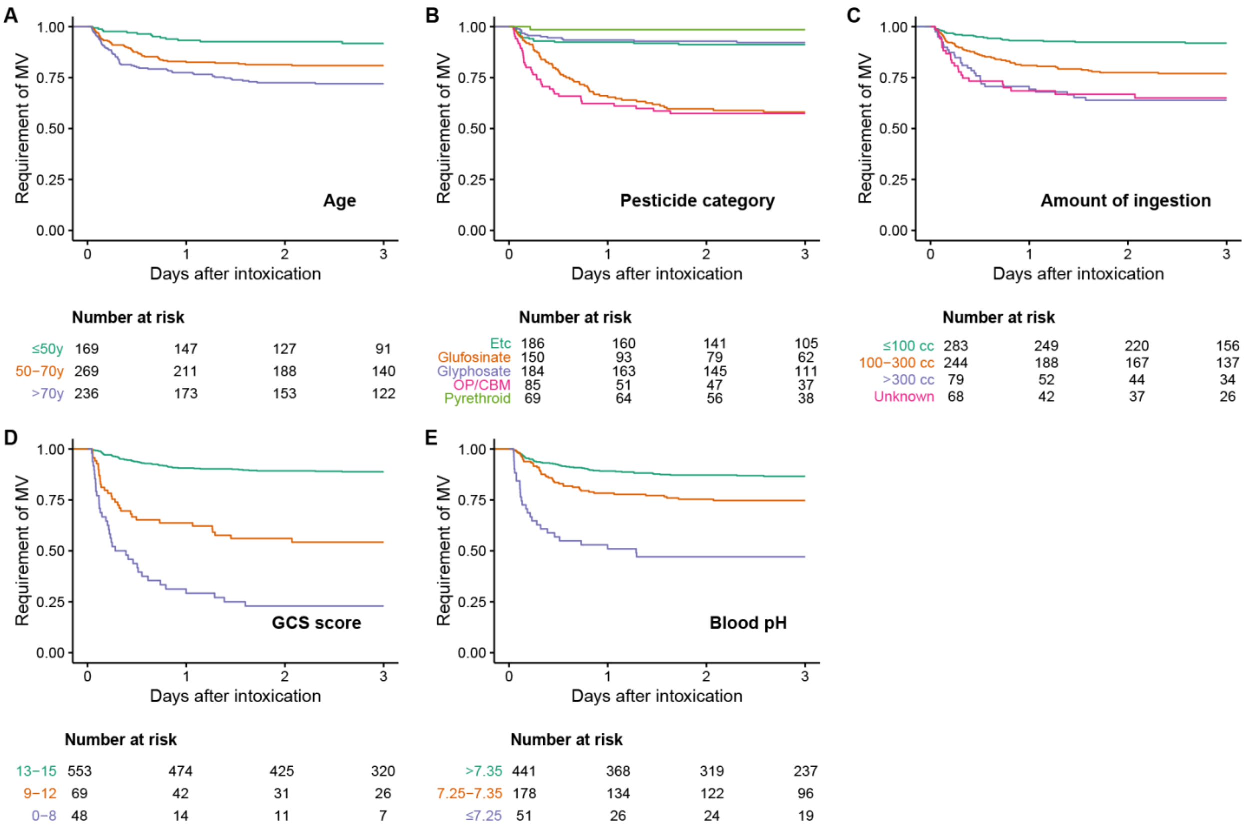

Figure S1. Kaplan-Meier curves for the mechanical ventilation requirements according to the categories of each predictor. The predictors for the Kaplan-Meier curves included age (A), pesticide category (B), amount of ingestion (C), GCS score (D), and arterial pH (E). OP, organophosphate; CBM, carbamate; GCS, Glasgow Coma Scale.

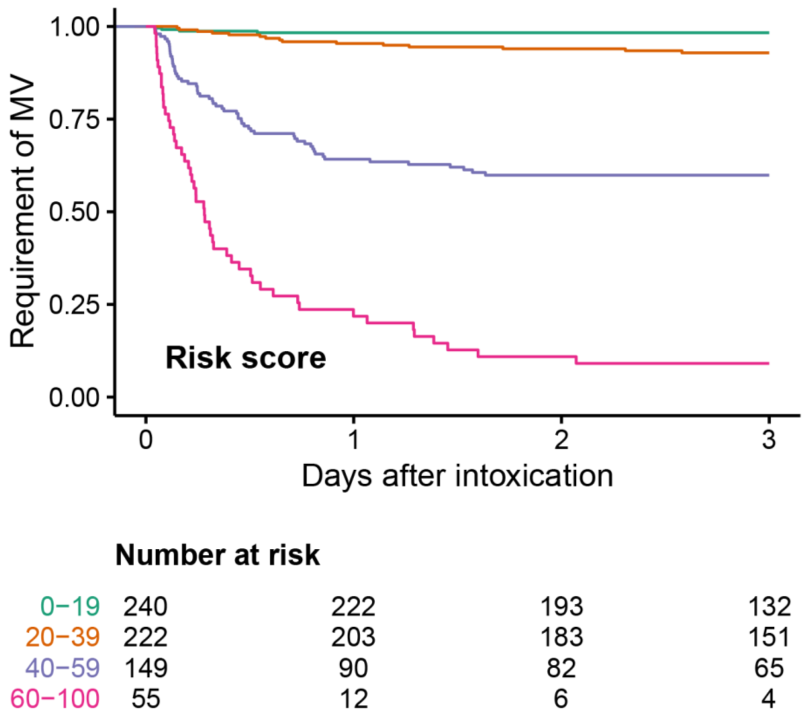

Figure S2. Kaplan-Meier curve for the mechanical ventilation requirement according to the risk categories of the prediction model. The risk categories were divided into four groups: 0-19, 20-39, 40-59 and 60-100 points.
